# Editor's Note to ‘Transcription activation by targeted recruitment of the RNA polymerase II CTD phosphatase FCP1’

**DOI:** 10.1093/nar/gkac622

**Published:** 2022-07-15

*Nucleic Acids Research*, Volume 29, Issue 17, 1 September 2001, Pages 3539–3545, https://doi.org/10.1093/nar/29.17.3539

In November 2021, a reader raised concerns about potential image manipulation in Figure 3A. The authors are unable to provide the original data generated more than 20 years ago. The editors and the authors’ institution have since examined the image, and the results were inconclusive. The editors advise readers to consider this image with care.

Julian E. Sale and Barry L. Stoddard

Senior Executive Editors

